# Maternal bacteremia in intrapartum fever: the role of ampicillin resistance and prolonged membrane rupture—a retrospective comparative study

**DOI:** 10.1007/s00404-025-08030-6

**Published:** 2025-04-23

**Authors:** Raneen Abu Shqara, Omer Saporta, Daniel Glikman, Lior Lowenstein, Maya Frank Wolf

**Affiliations:** 1 Department of Obstetrics & Gynecology, Raya Strauss Wing of Obstetrics and Gynecology, Galilee Medical Center, PO Box 21, 22100 Nahariya, Israel; 2 Azrieli Faculty of Medicine, Bar Ilan University, Safed, Israel

**Keywords:** Ampicillin, Bacteremia, Chorioamnionitis, Neonatal morbidity, Intrapartum fever, Enterobacteriaceae

## Abstract

**Objective:**

Intrapartum fever (IPF) (≥ 38.0 °C), if treated inappropriately, can lead to maternal bacteremia. In a cohort of women with IPF, we investigated perinatal, obstetrical, and microbiological outcomes, comparing those with bacteremia to those with negative blood cultures.

**Methods:**

A retrospective cohort study at a tertiary hospital (2010–2022) focused on women attempting vaginal delivery who were diagnosed with IPF. Outcomes were compared between those with bacteremia vs. negative blood cultures. After delivery, chorioamniotic swab cultures were obtained. Bacterial distribution and rates of ampicillin-resistant Enterobacteriaceae in blood and swab cultures were described. Women with Group B streptococcal colonization or prolonged rupture of membranes (ROM) received prophylactic ampicillin. The results were compared using univariate and multivariate analysis.

**Results:**

Overall, 78 women had bacteremia, and 341 had negative blood cultures. Women with bacteremia had higher rates of endometritis (*p* = 0.016), Apgar-5 < 7 (*p* = 0.021) and umbilical cord pH < 7.1 (*p* = 0.008). In multivariate analysis, prolonged ROM (*p* = 0.028) and prophylactic ampicillin (*p* = 0.036) were linked to maternal bacteremia. Maternal bacteremia (*p* < 0.001) was associated with higher endometritis and NICU admission rates. Blood cultures and chorioamniotic swab cultures matched in 65.9% of cases. Ampicillin-resistant Enterobacteriaceae spp. were found in 70.2% of blood cultures and 90.6% of chorioamniotic swab cultures. The rate of Enterobacteriaceae-isolated maternal bacteremia was higher among preterm than term deliveries (*p* = 0.034); while the rate of GBS-isolated bacteremia was lower (*p* < 0.001).

**Conclusion:**

Ampicillin-resistant Enterobacteriaceae rates in blood and chorioamniotic swab cultures were concerning. Prolonged ROM and prophylactic ampicillin were associated with higher maternal bacteremia rates. Appropriate use of intrapartum antibiotics is essential.

## What does this study add to the clinical work

**Table:** 

This study highlights the high prevalence of ampicillin-resistant Enterobacteriaceae as a cause of maternal peripartum bacteremia. It identifies prolonged ROM and prophylactic ampicillin as risk factors for maternal bacteremia, emphasizing the need for judicious antibiotic use.	

## Introduction

The prevalence of intrapartum fever (IPF), defined as a body temperature ≥ 38.0 °C, varies widely [1, 2]. IPF may be associated with an infectious or noninfectious etiology, such as epidural analgesia and the use of prostaglandins. Risk factors for IPF include prolonged labor, prolonged rupture of membranes (ROM), preterm prelabor ROM (PPROM) and nulliparity [3].

True maternal sepsis is rare; only an estimated 1.4% of pregnant patients with term clinical chorioamnionitis develop severe sepsis [1]. Sepsis is currently defined as life-threatening organ dysfunction caused by a dysregulated host response to infection [4]. As the gold standard for diagnosing sepsis in pregnancy has not been determined, the diagnosis of bacteremia, according to a positive blood culture, might identify patients at risk of adverse outcomes [5–7]. Ashwal et al. reported a 4.5% rate of maternal bacteremia among patients with IPF [8].

A few studies focused on the microbiological origin of intrapartum bacteremia [9–11]. Enterobacteriaceae-associated bacteremia was reported to occur predominantly in the third trimester of pregnancy and was most frequently associated with urinary infections, followed by genital ascending infections [9]. Rates of prematurity were substantially higher among patients with Gram-negative than Gram-positive isolated bacteremia [11].

Despite substantial advances in medical practice, maternal sepsis remains a major and potentially preventable cause of maternal mortality and morbidity worldwide [12]. Among patients with IPF and available blood culture results, we aimed to identify risk factors associated with bacteremia and to examine associations between maternal bacteremia and adverse perinatal and obstetrical outcomes. In addition, we sought to investigate the distribution of pathogens in chorioamniotic swab cultures in patients with positive and negative blood culture results, and to compare the rates of maternal bacteremia between patients with PPROM and preterm labor.

## Methods

### Study population

This retrospective study included pregnant patients who attempted vaginal delivery in a tertiary university-affiliated hospital between January 2010 and January 2022. The Institutional Review Board of the Galilee Medical Center approved the study. Included were pregnant patients with a singleton pregnancy who attempted vaginal delivery, with a fever > 38 °C and an available blood culture result. Exclusion criteria were multiple pregnancy, major fetal anomalies, intrauterine fetal death and the absence of an available blood culture or other missing data. The data were extracted from our electronic medical records and a chart review was performed.

### Outcome measures

Chorioamnionitis was defined as a maternal temperature greater than or equal to 39.0 °C, or maternal temperature of 38.0–38.9 °C and one additional clinical risk factor, such as maternal leukocytosis > 15,000/mm^2^, purulent cervical drainage and fetal tachycardia (> 160 beats/min) [13].

Endometritis was defined based on a fever ≥ 38 °C in the absence of any other cause, together with an associated clinical finding such as uterine tenderness, purulent lochia, tachycardia or abdominal pain. Early-onset sepsis (EOS) was defined as culture-proven sepsis during the 7 days from birth. PPROM was defined as ROM prior to 37 weeks of pregnancy. PPROM antibiotic treatment consisted of intravenous ampicillin (2 g every 6 h for 48 h), followed by oral amoxicillin (500 mg every 8 h) for an additional five days, and simultaneously with oral roxithromycin (150 mg twice daily for 7 days).

The study had several objectives: (a) to compare the co-primary outcomes of puerperal endometritis and neonatal intensive care unit (NICU) admission between patients with positive and negative blood cultures (b) to identify clinical and obstetrical risk factors for bacteremia in patients with intrapartum fever using multivariate analysis, and (c) to describe the microbiological and susceptibility patterns of pathogens isolated from blood and chorioamniotic swab cultures, with a focus on identifying antibiotic-resistant organisms, particularly ampicillin-resistant Enterobacteriaceae.

Secondary maternal outcomes consisted of cesarean section, intensive care unit (ICU) admission and postpartum hemorrhage. Secondary neonatal outcomes included 5-min Apgar score < 7, umbilical artery pH < 7.1, meconium aspiration syndrome, respiratory distress syndrome (RDS) and the need for ventilation support. Among patients who had a preterm delivery, we compared bacteremia rates between those with PPROM and with preterm labor.

During the study period, the routine clinical practice for our patients with body temperature > 38 °C was to obtain a chorioamniotic membrane swab culture. After its extraction, the placenta was placed on a sterile surface, and the chorioamniotic space was separated with sterile gloves and sampled with sterile swab cultures [14]. The results of the blood cultures and chorioamniotic membrane swabs and antimicrobial susceptibility tests were reviewed by an infectious disease specialist, and certain bacteria were defined as contaminants. Patients who were known group B streptococcus (GBS) carriers and those with ROM ≥ 18 h and spontaneous preterm labor received intrapartum ampicillin 2 g four times a day. According to our department protocol, during the study period, blood cultures were obtained from patients with IPF. Specifically, in those with signs of clinical chorioamnionitis, blood cultures were drawn before the initiation of intravenous ampicillin (2 g q.i.d) and gentamicin (240 mg q.d) [15].

### Sample size calculation

NICU admission following IPF and negative maternal blood culture was previously reported as 51.1% [16]. An effect size of 20% change in the NICU admission rate was considered significant, alpha 0.05, power of 90%, 2-sided. Accordingly, the sample size required was at least 246. Our sample exceeded that threshold.

### Statistical analysis

Continuous variables are presented as means and standard deviations or medians and ranges. Qualitative data are shown as frequencies and percentages. Continuous variables were compared using the independent sample t-test or Mann–Whitney test. Categorical variables were analyzed using Pearson’s chi-squared test or Fisher’s exact test. Multivariate regression models were used to predict bacteremia, endometritis, and NICU admission, controlling for relevant variables. Statistical analysis was performed using IBM SPSS Statistics for Windows, version 27.0 (IBM Corp., Armonk, NY, USA).

## Results

During the study period, 54,132 patients gave birth in our medical center, of whom 601 were diagnosed with IPF (1.1%). Of those, 182 were excluded from the analysis: due to the absence of a blood culture (*n* = 138), records with missing data (*n* = 22), multiple pregnancies (*n* = 15) and fetal anomalies (*n* = 7). The final sample consisted of 419 patients, 78 with positive blood cultures and 341 with negative blood cultures.

The patients’ characteristics are shown in Table 1. For the patients with positive versus negative blood cultures, the mean parity was higher: 2.1 ± 1.7 vs. 1.6 ± 1.2, *p* = 0.026, and the rates were lower of cervical ripening by PGE2 and epidural analgesia: 3.8% vs. 12.3%, *p* = 0.025; and 52.6% vs. 75.4%, *p* < 0.001, respectively. The co-primary outcomes, namely, the puerperal endometritis rate and admission to NICU, were significantly higher among patients with positive vs. negative blood cultures. Endometritis rates were 16.7% vs. 7.9% (*p* = 0.016), while NICU admission rates were 50.0% vs. 37.5% (*p* = 0.042).

**Table 1:** Maternal characteristics

	Negative blood culture *N* = 341	Positive blood culture *N* = 78	*p*-value
Maternal age, years	27.4 ± 4.9	28.5 ± 1.7	0.095
Delivery number	1.6 ± 1.2	2.1 ± 1.7	0.026
Assisted reproductive therapy	23 (6.7)	6 (7.7)	0.804
Diabetes mellitus^a^	34 (9.8)	8 (10.2)	0.939
Cervical ripening by catheter balloon	45 (13.2)	14 (17.9)	0.281
Cervical ripening by PGE2	42 (12.3)	3 (3.8)	0.025
Epidural anesthesia	257 (75.4)	41 (52.6)	< 0.001

The data are presented as mean ± standard deviation or as number (percentage)

^a^Including pregestational diabetes and gestational diabetes mellitus

PGE2- prostaglandin E2

### Secondary maternal findings:

**Fig. 1:**
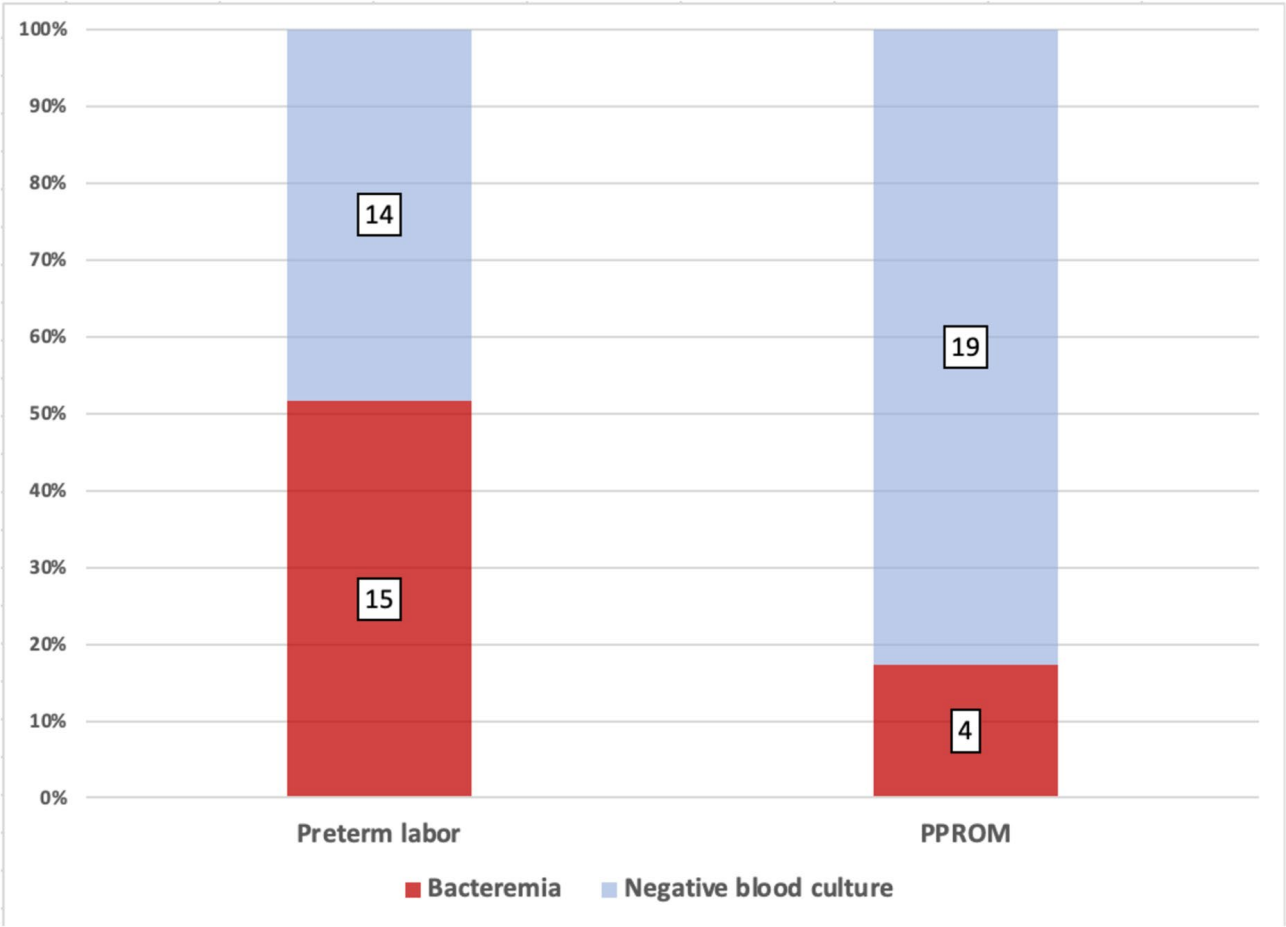
Rates of bacteremia in preterm intrapartum fever, sub-analyzed to PPROM and preterm labor. PPROM- preterm prelabor rupture of membranes

Among patients with positive vs. negative blood cultures, the rates were higher of: clinical chorioamnionitis, 39 (50%) vs. 121 (35.5%), *p* = 0.017; intrapartum ampicillin treatment, 56 (71.8%) vs. 143 (41.9%), *p* < 0.001; and prolonged ROM, 43 (55.1%) vs. 91 (26.6%), *p* < 0.001 (Table 2). The rates were also higher for preterm birth at < 37 weeks and < 34 weeks: 19 (24.4%) vs. 33 (9.7%) and 9 (11.5%) vs. 10 (2.9%), *p* < 0.001 for both. Rates of GBS carrier status, PPROM and cesarean delivery were similar. Rates of maternal ICU admission were higher among patients with positive vs. negative blood cultures: 6 (7.7%) vs. 1 (0.29%), *p* < 0.001. No incidences of maternal mortality occurred. The rates of postpartum hemorrhage were similar. The median (range) hospitalization length was longer in the positive blood culture group: 10 (5–32) vs. 4 (2–27), *p* < 0.001. The rate of bacteremia was lower among patients with PPROM (*n* = 23) who received antibiotic prophylaxis with ampicillin and azithromycin than among patients with preterm labor (*n* = 29) who received ampicillin alone, 4 (17%) vs. 15 (51.7%), *p* = 0.019 (Fig. 1).

**Table 2:** Obstetrical and perinatal findings

	Negative blood culture *N* = 341	Positive blood culture *N* = 78	p-value
*Obstetrical findings*			
Clinical chorioamnionitis	121 (35.5)	39 (50)	0.017
Delivery week	39.3 ± 2.1	37.6 ± 3.3	< 0.001
Preterm birth < 37 weeks	33 (9.7)	19 (24.4)	< 0.001
Preterm birth < 34 weeks	10 (2.9)	9 (11.5)	< 0.001
Ampicillin prophylaxis during delivery	143 (41.9)	56 (71.8)	< 0.001
GBS carrier	70 (20.6)	22 (28.2)	0.139
Prolonged ROM	91 (26.6)	43 (55.1)	< 0.001
PPROM	19 (5.6)	4 (5.1)	0.787
Delivery method			
Normal delivery	180 (52.8)	38 (48.7)	0.516
Cesarean delivery	161 (47.2)	40 (51.3)	
ICU admission	1 (0.29)	6 (7.7)	< 0.001
Postpartum hemorrhage	12 (3.5)	3 (3.8)	1.000
Puerperal endometritis	27 (7.9%)	13 (16.7)	0.016
Hospitalization length	4 (2–27)	10 (5–32)	< 0.001
*Neonatal findings*			
Neonatal weight, g	3 351 ± 501	3 130 ± 703	0.001
Weight in preterm, g	2 731 ± 784	2 220 ± 719	0.023
Weight in term delivery, g	3 421 ± 410	3 423 ± 371	0.971
Apgar 5 ≤ 7	4 (1.1)	4 (5.1)	0.021
Umbilical cord pH < 7.1	3 (0.9)	4 (5.1)	0.008
NICU admission	128 (37.5)	39 (50.0)	0.042
Neonatal sepsis	7 (2.1)	3 (3.9)	0.349
Total cohort neonatal hospitalization length, days	3 (2–103)	5 (3–38)	< 0.001
Term neonatal hospitalization length, days	3 (2–32)	4 (3–29)	< 0.001
Need for respiratory support	28 (8.2)	15 (15.4)	0.006
Respiratory distress syndrome	8 (2.3)	6 (7.7)	0.029
Necrotizing enterocolitis	1 (0.3)	1 (1.3)	0.338
Meconium aspiration syndrome	5 (1.5)	1 (1.3)	1.000

The data are presented as mean ± standard deviation, as median (range) or as number (percentage)

GBS- group B streptococcus, ROM- rupture of membranes, PPROM- premature prelabor rupture of membranes, ICU- intensive care unit, NICU- neonatal intensive care unit

### Secondary neonatal findings

The median neonatal weights in the total cohort and in preterm were lower among patients with positive than negative blood cultures: 3 130 + 703 vs. 3 351 ± 501, *p* = 0.001 and 2 220 ± 719 vs. 2 731 ± 784, *p* = 0.023, respectively (Table 3). Weight at term deliveries were similar. Rates of Apgar 5 ≤ 7 and umbilical cord pH < 7.1 were higher in the positive vs. negative blood culture groups: 4 (5.1%) vs. 4 (1.1%), *p* = 0.021 and 4 (5.1%) vs. 3 (0.9%), *p* = 0.008. Additionally, the need for respiratory support (15.4% vs. 8.2%, *p* = 0.006) and rates of RDS (7.7% vs. 2.3%, *p* = 0.029) were increased in the positive blood culture group. Neonatal hospitalization length in the total cohort and among patients who delivered at term were longer in the positive vs. negative blood culture group, 5 (3–38) vs. 3 (2–103), *p* < 0.001 and 4 (3–29) vs. 3 (2–32), *p* < 0.001, respectively. Rates of neonatal sepsis, necrotizing enterocolitis and meconium aspiration syndrome were similar between the groups. No incidences of neonatal encephalopathy occurred.

**Table 3:** Multivariate analysis

	Odds ratio	95% confidence interval	*p*
*Model 1: Multivariate analysis model for predicting maternal bacteremia among patients with IPF*			
Maternal age, years	1.02	0.97–1.08	0.399
Parity	0.91	0.74–1.13	0.410
Cervical ripening by catheter balloon	1.34	0.62–2.89	0.451
Cervical ripening by PGE2	0.15	0.04–0.57	0.005
GBS carrier	1.43	0.54–3.82	0.474
Preterm delivery < 37 weeks	1.88	0.9–3.91	0.092
Prolonged ROM ≥ 18 h	3.39	1.14–10.08	0.028
Ampicillin treatment during delivery	1.33	1.15–4.65	0.036
Epidural anesthesia	0.27	0.15–0.49	< 0.001
*Model 2: Multivariate analysis model for predicting endometritis among patients with IPF*			
Positive vs. negative maternal blood culture	1.67	1.21–4.15	< 0.001
Maternal age, years	0.96	0.87–1.06	0.440
Parity	0.95	0.63–1.45	0.824
Cervical ripening by catheter balloon	0.615	0.20–2.60	0.720
Cervical ripening by PGE2	1.23	0.34–4.47	0.753
GBS carrier	0.43	0.12–1.54	0.196
Preterm delivery < 37 weeks	1.99	0.60–6.55	0.260
Prolonged ROM ≥ 18 h	2.17	1.04–5.31	0.046
Ampicillin treatment during delivery	0.54	0.06–4.79	0.578
Cesarean delivery	4.97	1.90–13.05	0.001
*Model 3: Multivariate analysis model for predicting NICU admission among neonates of patients with IPF*			
Positive vs. negative maternal blood culture	1.85	1.08–3.18	< 0.001
Preterm birth < 37 weeks	2.38	1.12–5.06	0.024
Birthweight < 2 500 g	1.69	0.54–5.28	0.369
Prolonged ROM	0.81	0.33–2.04	0.662
Cervical ripening by catheter balloon	0.91	0.52–1.60	0.737
GBS carrier	0.92	0.77–6.12	0.851
Intrapartum ampicillin	2.17	1.10–7.10	0.141

IPF, intrapartum fever; PGE2, prostaglandin E2; NICU, neonatal intensive care unit; ROM, rupture of membranes; RDS, respiratory distress syndrome; GBS, group B streptococcus

### Multivariate analysis

Table 3 presents the results of the multivariate logistic regression models. Cervical ripening by PGE2 (odds ratio (OR) 0.15, 95%, CI 0.04–0.57), and epidural analgesia (OR 0.27, CI 0.15–0.49, *p* < 0.001) were independently associated with lower rates of positive vs. negative blood cultures. Prolonged ROM ≥ 18 h (OR 3.39, CI 1.14–10.08, *p* = 0.028), and intrapartum ampicillin (OR 1.33, CI 1.15–4.65, *p* = 0.036) were independently associated with higher rates of positive vs. negative blood cultures. A positive maternal blood culture result (OR 1.67, CI 1.21–4.15, *p* < 0.001), prolonged ROM (OR 2.17, CI 1.04–5.31, *p* = 0.046), and cesarean delivery (OR 4.97, CI 1.90–13.05, *p* = 0.001) were independently associated with higher rates of endometritis.

A positive maternal blood culture result (OR 1.85, CI 1.08–3.18, *p* < 0.001) and preterm birth (OR 2.38, CI 1.12–5.06, *p* = 0.024) were independently associated with higher rates of NICU admission.

### Microbiological findings

**Fig. 2:**
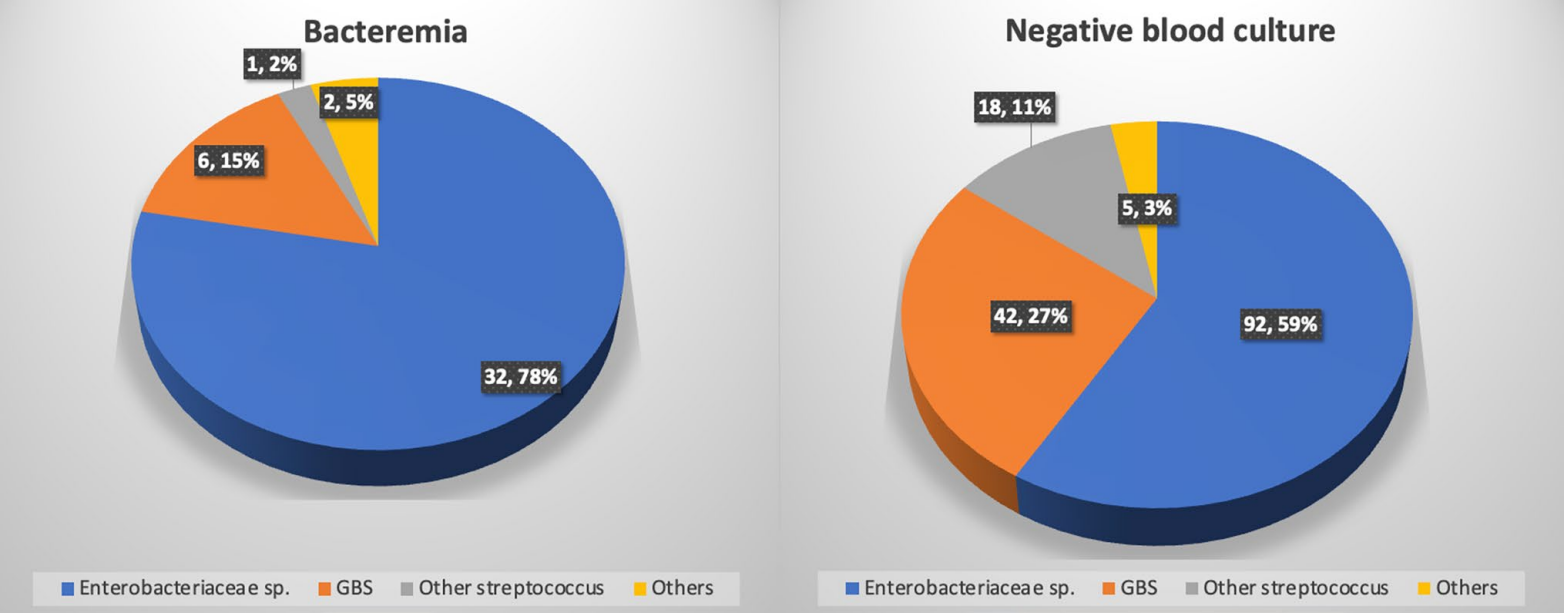
Bacterial distribution in chorioamniotic swab cultures. Others: including enterococcus Faecalis and anaerobes

Pathogen distribution in patients with a positive blood culture was the following: Enterobacteriaceae sp (*n* = 47, 60%), GBS (*n* = 17, 22%), other streptococci species (*n* = 8, 10%) and other pathogens (*n* = 6, 8%). Among the 47 Enterobacteriaceae isolates identified in blood cultures, 33 (70.2%) were resistant to ampicillin. Resistance was also observed to trimethoprim/sulfamethoxazole in 11 isolates (23.4%), third-generation cephalosporins in 8 (17.0%), fluoroquinolones in 4 (8.5%), and aminoglycosides in 4 (8.5%). None of the isolates were resistant to carbapenems. Among patients with positive vs. negative blood cultures, a higher rate of Enterobacteriaceae sp. isolates was found in the chorioamniotic swab cultures: 32 (78.0%), vs. 92 (59.0%), *p* = 0.021. Among them, 27 (84.3%) vs. 62 (67.4%), *p* = 0.0379 were ampicillin-resistant (Fig. 2). Rates of other pathogens including GBS, were similar. Positive blood cultures and chorioamniotic swab cultures matched in 65.9% of the patients. In those with positive blood cultures, the rate of Enterobacteriaceae-isolated bacteremia was higher among those with preterm than term deliveries, 14/19 (73.7%) vs. 28/59 (45.8%), *p* = 0.034; while the rate of GBS-isolated bacteremia was lower, 0 (0%) vs. 18 (30.5%), *p* < 0.001.

## Discussion

### Main findings

Among patients with positive blood cultures, rates were higher of endometritis, preterm delivery, maternal ICU admission, neonatal intensive care admission, RDS and cord pH < 7.1. In multivariate analysis, correlations of positive blood culture with endometritis and NICU admission remained significant. Additionally, prolonged ROM and intrapartum prophylactic ampicillin were correlated with higher rates of positive blood cultures. Positive blood cultures and chorioamniotic swab cultures matched in 65.9% of the patients. Among those with positive blood cultures, rates of ampicillin-resistant Enterobacteriaceae in blood cultures and chorioamniotic swab cultures were 70.2% and 84.3%, respectively. Among patients with positive blood cultures, for those with preterm versus term deliveries, the rate of Enterobacteriaceae-isolated bacteremia was higher, and of GBS-isolated bacteremia lower.

### Maternal outcomes

We report correlations of non-infectious causes for IPF, such as epidural anesthesia and PGE2, with negative rather than positive blood cultures. Previously, among patients who received epidural analgesia, higher rates of IPF were reported for those nulliparas versus multiparous (11%–33% vs. 4%) [1, 17]. As PGE_2_ is well established as the final mediator of fever, it is listed among the non-infectious cases of IPF [18]. Previously, the incidence rate of peripartum ICU admission was reported to be 0.1%. In our cohort of patients with positive blood cultures, this rate was significantly higher at 7.7% [19]. Intrapartum ampicillin prophylaxis was associated with positive blood cultures among patients with IPF. In our study, the ampicillin was started prior to the fever, due to GBS colonization or prolonged ROM. This aligns with the findings of Attali et al., who reported that patients who developed maternal bacteremia had a significantly higher rate of antibiotic prophylaxis administered before the onset of fever compared to those without bacteremia [20]. We previously reported that the use of ampicillin during delivery might promote ampicillin-resistant *E. coli* growth in the chorioamniotic membranes and consequently lead to higher rates of infectious morbidity [16]. Our findings suggest that early intrapartum antibiotic administration, although aimed at prophylaxis, may contribute to antimicrobial resistance, potentially leading to more severe peripartum infections.

In the current study, we found that patients with IPF and positive blood culture presented at an earlier gestational age. The role of maternal infection in preterm birth is well-described [21, 22]. According to a Brazilian multicenter study, about 65.9% of all preterm births were associated with maternal infection [22]. Furthermore, we found lower rates of positive maternal blood culture among patients with PPROM who received dual antibiotic prophylaxis than among patients with spontaneous preterm birth who received ampicillin alone as antibiotic prophylaxis (intact membranes). This implies that extended antibiotics prophylaxis in preterm labor might lower the rates of maternal bacteremia. However, to verify this, a larger sample size and a randomized controlled trial setting are needed.

### Neonatal outcomes

In the current study, we showed that maternal bacteremia during delivery was associated with adverse perinatal outcomes including NICU admission, RDS, the need for respiratory support, and longer hospitalization length. Although we did not find correlations of bacteremia with neonatal sepsis and meconium aspiration, our sample was not powered for these results. A recently published study demonstrated a significant correlation between maternal bacteremia and culture-proven EOS, with 3.9% of infants born to bacteremic mothers developing EOS compared to none among infants of non-bacteremic mothers [23]. Our finding of a correlation between positive maternal blood culture and preterm birth is consistent with a previous study, which demonstrated a significant association between maternal peripartum sepsis and preterm delivery [24]. In previous studies, intrapartum maternal infection was associated with meconium aspiration syndrome, hyaline membrane disease, neonatal seizures and assisted ventilation [25, 26]. We reported higher rates of umbilical artery pH < 7.1 and Apgar 5 < 7. However, no incidences of neonatal encephalopathy occurred. On the other hand, among patients with IPF, Impey et al. [1] reported a rate of neonatal encephalopathy of 2.1%. Others reported that the rates of encephalopathy or the need for therapeutic hypothermia were higher among patients with a body temperature of > 39 °C, compared to 38–39 °C (1.1 vs 4.4%). The worrisome increase in neonatal adverse outcomes in patients with bacteremia indicates that preventive measures for the occurrence of bacteremia should be incorporated, such as appropriate antibiotic use and earlier treatment of suspected chorioamnionitis.

### Microbiological outcomes

The reported 65.9% match between the chorioamniotic swab culture results and blood cultures indicates that most incidences of bacteremia were probably due to chorioamnionitis. The risk of bacteremia in chorioamnionitis was reported as 5–10% [27]. *E. coli* is consistently cited as a major pathogen responsible for maternal sepsis during pregnancy, causing 33–50% of the occurrences and 10–27% of the fetal mortality [24, 28].

Our findings are novel, as they highlight a shift in the bacterial profile of maternal bacteremia [23], with Enterobacteriaceae predominating in preterm deliveries and GBS in term deliveries. These findings reflect a pattern similar to the bacterial distribution seen in EOS [29], where Gram-negative organisms are more prevalent in preterm infants. The predominance of Enterobacteriaceae in preterm deliveries highlights a potential gap in current prophylactic strategies, as ampicillin—commonly used for GBS prophylaxis in preterm labor [30, 31]—may not adequately cover Gram-negative organisms. These findings emphasize the need for future research to evaluate the effectiveness of broad-spectrum antibiotics in reducing infection-related morbidity in preterm deliveries.

We report alarmingly high rates of ampicillin-resistant *Enterobacteriaceae* in both blood cultures and chorioamniotic swab cultures. Our results corroborate a conclusion derived from data over a decade that showed an increase of ampicillin-resistant *Enterobacteriaceae* (up to 81%) in placental cultures [32]. The rise in antimicrobial resistance among patients with IPF highlights the need to examine the epidemiology, risk factors, and preventive measures of Enterobacteriaceae-related infections, including appropriate antibiotic use. [33, 34].

### Strengths and limitations

The main strength of this study is the inclusion of blood culture and chorioamniotic swab cultures for all the patients with IPF. The limitations include its retrospective nature. Although we retrieved data from over a decade, the study was not powered for rare outcomes such as EOS and meconium aspiration syndrome. Furthermore, contamination of the cultures of the chorioamniotic surface after a vaginal birth could not be ruled out. Although institutional guidelines remained consistent throughout the study period, 23% of patients with IPF did not have available blood cultures. This may be attributed to a low level of clinical suspicion for infection or clinician discretion at the time of care. Importantly, the rate of patients without blood cultures remained stable over the 12-year period, suggesting consistent clinical practice patterns. However, this could introduce bias, as missed or delayed diagnoses of bacteremia in these patients may have resulted in under-treatment or complications that were not captured in our analysis. Lastly, we acknowledge that complete sterility during chorioamniotic swab collection could not be guaranteed, particularly following vaginal delivery, and the risk of sample contamination cannot be ruled out.

## Conclusions

Maternal bacteremia was associated with adverse maternal and perinatal complications. We report alarmingly high rates of ampicillin-resistant Enterobacteriaceae in both blood cultures and chorioamniotic swab cultures. Intrapartum-ampicillin treatment was associated with higher rates of maternal bacteremia. Appropriate use of intrapartum antibiotics is justified.

## Data Availability

No datasets were generated or analysed during the current study.
